# Dataset in support of the generation of Niemann-Pick disease Type C1 patient-specific iPS cell lines carrying the novel NPC1 mutation c.1180T>C or the prevalent c.3182T>C mutation – Analysis of pluripotency and neuronal differentiation

**DOI:** 10.1016/j.dib.2017.03.042

**Published:** 2017-04-02

**Authors:** Franziska Peter, Michaela Trilck, Michael Rabenstein, Arndt Rolfs, Moritz J. Frech

**Affiliations:** Albrecht-Kossel-Institute for Neuroregeneration (AKos), University Medicine Rostock, Gehlsheimer Straße 20, 18147 Rostock, Germany

**Keywords:** Induced pluripotent stem cells, Niemann-Pick Type C1, Neuronal differentiation

## Abstract

Data presented in this article demonstrate the generation and characterization of two novel Niemann-Pick disease Type C1 (NPC1) patient-specific induced pluripotent stem cell (iPSC) lines, related to the research article Trilck et al. (Diversity of Glycosphingolipid GM2 and Cholesterol Accumulation in NPC1 Patient-Specific iPSC-Derived Neurons; Brain Res.; 2017; 1657:52-61. doi: 10.1016/j.brainres.2016.11.031). For reprogramming fibroblasts, carrying the novel homozygous mutation c.1180T>C and the prevalent homozygous mutation c.3182T>C, were used. Reprogramming into patient-specific iPSCs was induced by retroviral transduction of the transcription factors Sox2, Klf4, Oct4 and c-Myc, and confirmed according to their pluripotency. The iPSCs were subsequently differentiated into neural progenitor cells, which were terminally differentiated into functional neurons and glial cells. The generation of these cell lines provides further valuable tools to investigate pathogenic mechanism of NPC1 in human neuronal cells carrying different NPC1 mutations.

**Specifications Table**

**Table:** 

Subject area	Cell biology
More specific subject area	Patient specific induced pluripotent stem cells and neuronal derivatives
Type of data	Phase contrast and fluorescence images, graphs of current and voltage traces of patch clamp recordings
How data was acquired	Fluorescence microscopy was performed with Keyence Biozero 8000 and a Carl Zeiss LSM780 microscope.
	Whole cell Patch clamp experiments in the current and voltage clamp mode were performed with a HEKA EPC-10-double amplifier with the according PatchMaster software
Data format	analyzed
Experimental factors	Human fibroblasts were transduced to induced pluripotent stem cells by retroviral transfection of Sox2, Klf4, Oct4 and c-Myc
Experimental features	Reprogramming of fibroblasts by retroviral system. Determination of karyotype by Giemsa Trypsin banding. Detection of marker for pluripotency by alkaline phosphatase (AP) staining and immunocytochemistry in induced pluripotent stem cells, embryoid bodies and neural progenitor cells. Specific marker for neurons and glial cells were detected by immunocytochemistry. Recording of functional ion channels by patch clamp technique.
Data source location	Albrecht-Kossel-Institute for Neuroregeneration (AKos), University Medicine Rostock, Rostock, Germany
Data accessibility	Data is provided with this article

**Value of the data**

• Data in support of the generation of patient-specific iPSCs carrying novel NPC1 mutation.
• Data demonstrating the pluripotency of generated iPSCs and derived neural progenitors.
• Data showing differentiation of neural progenitors into neurons and glial cells.
• Data describing the establishment of human cell model applicable in NPC1 disease modeling and drug discovery.

## 1 Data

**Fig. 1:**
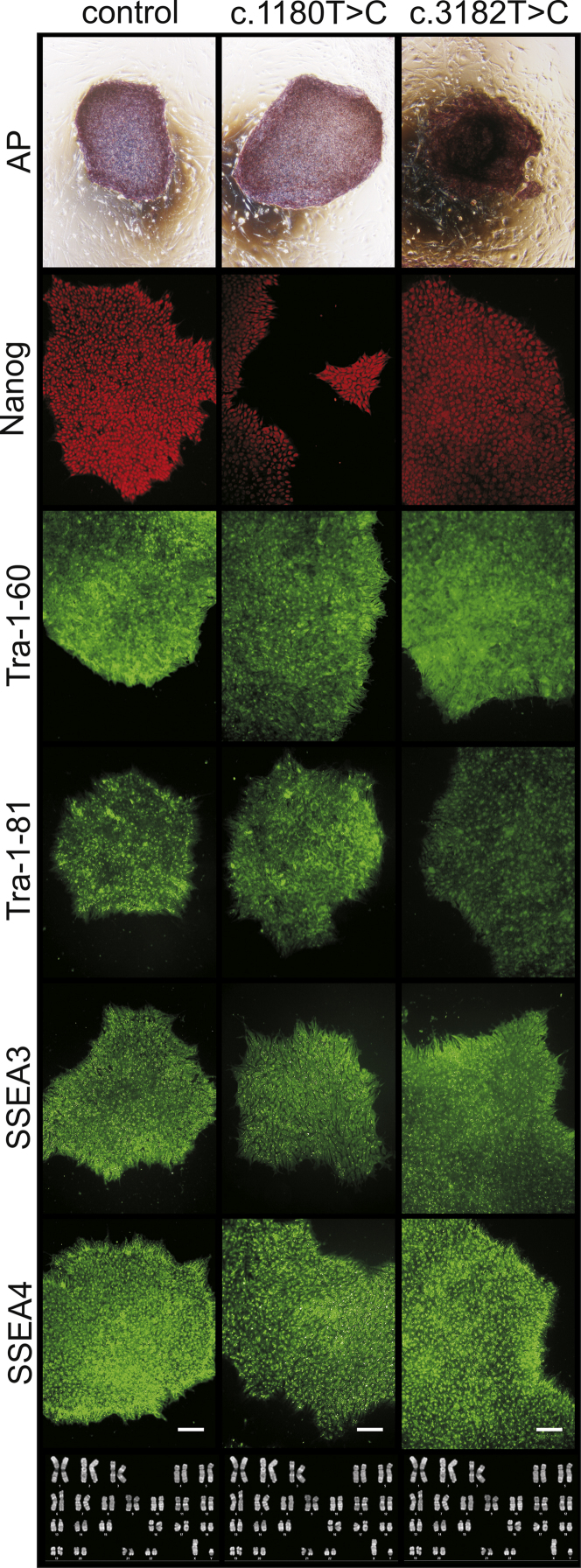
Examination of pluripotency of iPSCs and karyotype. Alkaline phosphatase (AP) staining (upper row). Photographs of immunocytochemistry, taken with a Keyence Biozero 800 microscope, show iPSC colonies being positive for pluripotency specific marker Nanog (red), Tra-1–60, Tra-1–81, SSEA3 and SSEA4 (all green). Scale bars in lower row are representative for all photographs and indicate 100 µm. Phase contrast picture of karyotype by Giemsa trypsin banding (last row) indicate no abnormalities due to reprogramming. No scale is given for karyotypes.

**Fig. 2:**
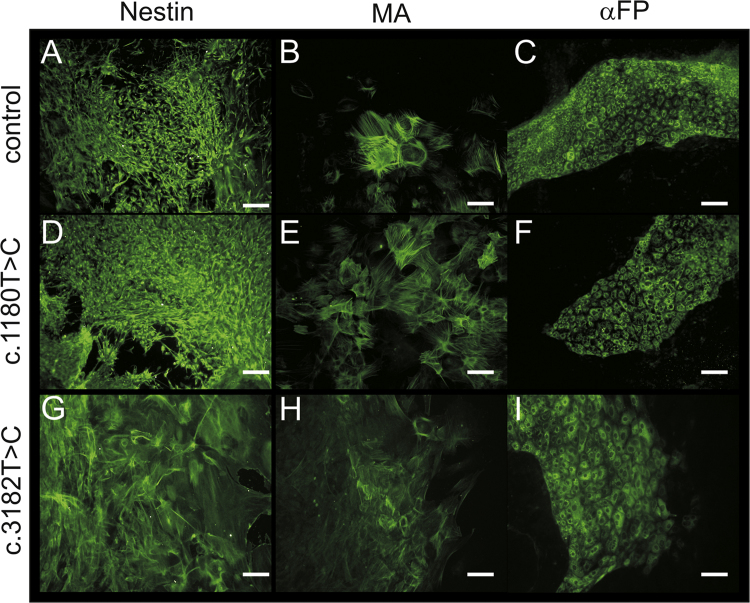
Tissue specific marker in iPSC-derived embryoid bodies. Immunofluorescence photographs of tissue specific markers Nestin (ectoderm), muscle actin (MA, mesoderm) and α-fetoprotein (αFP, endoderm). Scales show 100 µm.

**Fig. 3:**
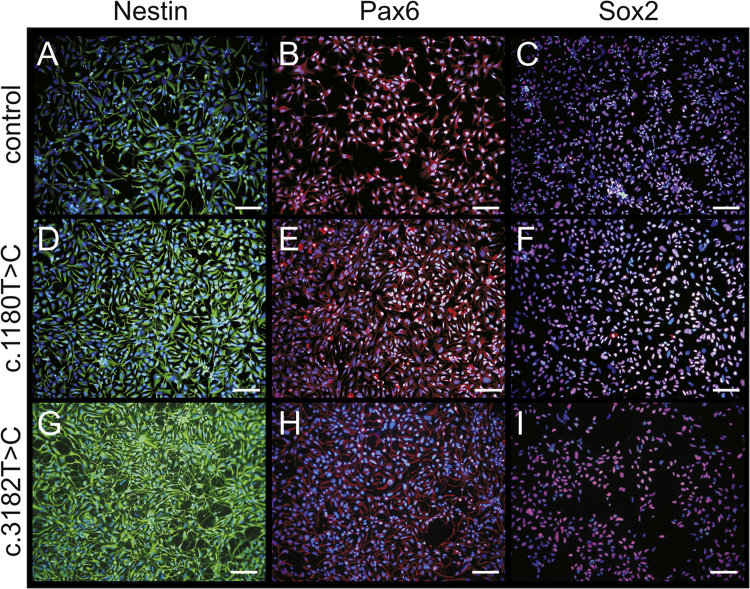
Examination of pluripotency of iPSCs derived neural progenitor cells. Neural progenitor cells were assessed by immunocytochemical analyses of the neural progenitor markers nestin (A,D,G, green), Pax6 (B,E,H, red) and Sox2 (C,F,I, red). Nuclei were counter stained with DAPI (blue). Scale show 100 µm. All pictures represent an overlay of fluorescence of immunocytochemical staining and DAPI staining.

**Fig. 4:**
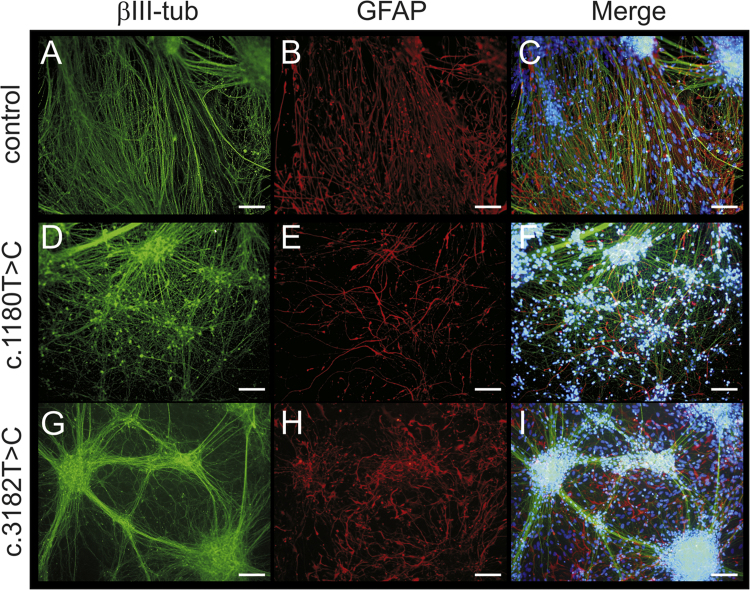
Examination of neuronal and glial marker in terminally differentiated cells. Neural progenitor cells were terminally differentiated into neurons positive for βIII-tub (A,D,G, green) and into GFAP-positive glial cells (B,E,H, red). Cells were differentiated for 6 weeks. An overlay is shown in the right panel (C,F,I). Nuclei were counter stained with DAPI (C,F,I, blue). Scale show 100 µm for all pictures.

**Fig. 5:**
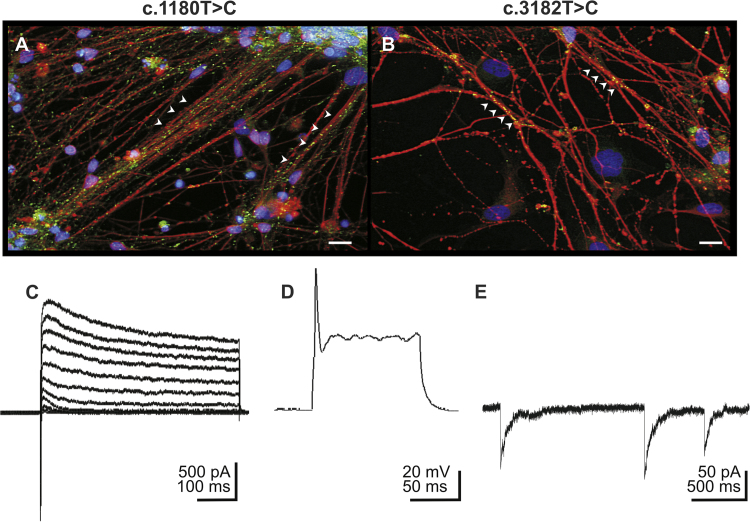
Examination of neural progenitor cells derived neurons. Neural progenitor cells were differentiated into neurons positive for βIII-tub (red in A and B) and the pre-synaptic marker synaptophysin (green in A and B, arrowheads, scale 20 µm). Nuclei were stained with DAPI (blue in A and B). Patch clamp recordings (examples are taken from cells carrying the c.1180T>C mutation) of neurons revealed functional voltage gated ion channels, demonstrated by inward-directed sodium currents and outward-directed potassium currents (C), as well as elicited action potentials (D). Spontaneous postsynaptic currents proved the maturation of a functional synaptic network (E).

These data show the generation of NPC1 patient-specific induced pluripotent stem cells (iPSCs) using a standardized protocol [1]. Data presented in this article are related to the research article Trilck et al. [2]. Fibroblasts of NPC1 patients, carrying the homozygous *NPC*1 mutation c.1180T>C or c.3182T>C, were used for reprograming. The c.1180T>C is a novel unpublished mutation while mutation c.3182T>C is the most prevalent mutation of the *NPC*1 gene found in patients [3]. Data of the new cell lines were compared with a control cell line for examination of differences in reprogramming and pluripotency which might occur due to *NPC*1 mutation. Data demonstrate the pluripotency of the generated iPSCs by immunocytochemical detection of a panel of specific pluripotency marker (Fig. 1). Pluripotency was further shown by induction of embryoid bodies and the immunocytochemical detection of nestin, muscle actin and α-feto protein (αFP), specific markers for the three germ layers (Fig. 2). Data present the differentiation of iPSCs into neural progenitor cells (Fig. 3). Terminal neuronal differentiation in neurons and in glial cells is shown by immunocytochemical detection of the neuron specific marker βIII-tubulin (βIII-tub) and synaptophysin (Fig. 5) and the glial cell specific marker GFAP (Fig. 4). Data obtained by patch clamp recordings show differentiation of neural progenitor cells into functional neurons (Fig. 5).

## 2 Material and methods

### 2.1 Cell culture

Human dermal fibroblast cell lines from male donors were obtained from Coriell Institute for Medical Research, Camden, USA (NPC1 homozygous mutated: c.3882T>C [p.I1061T], GM18453) and Centogene AG, Rostock, Germany (control and NPC1 homozygous mutated c.1180T>C [p.Y395H]), respectively. Cells were cultivated in fibroblast medium containing DMEM high glucose, 10% FBS and 1% penicillin/streptomycin. Mitotically inactivated mouse embryonic fibroblasts (Amsbio, United Kingdom) were used as the feeder cell layer for iPSCs and were cultivated in fibroblast medium. HEK293FT cells (Invitrogen, Germany) were also cultivated in fibroblast medium but without antibiotics. Human iPSCs were cultured on a feeder cell layer in iPSC medium containing DMEM/F12, 20% knockout serum replacement, 1% penicillin/streptomycin, 1% GlutaMAX, 1% MEM non-essential amino acids, 0.2% 2-mercaptoethanol, and 10 to 15 ng/ml FGF2 (Amsbio, United Kingdom) or on Matrigel (Corning, Netherlands) cultured in mTESR1 medium (Stemcell Technologies, France). Medium was changed daily and cells were passaged weekly. All cells were cultivated at 37 °C in a saturated humidity atmosphere containing 5% CO_2_.

### 2.2 Reprogramming of fibroblasts into iPSCs

Human iPSCs were generated as described earlier [1]. In brief, retroviruses were produced in HEK293FT cells using a retroviral vector encoding for GFP and one of the transcription factors (Sox2, Klf4, Oct4, or c-Myc) as well as VSV-G and Gag-Pol by XtremeGene 9 (Roche, Germany). Virus titering was performed through FACS analysis of the percentage of GFP-positive HEK293FT cells. The human fibroblasts were transduced with Sox2, Oct4, Klf4 (70–80% infection efficiency), and c-Myc (40–50% infection efficiency) in the presence of 5 µg/ml protamine sulfate (in fibroblast medium). After 48 h, cells were reseeded onto a gelatin-coated 6 cm-dish. The following day, medium was replaced with iPSC medium supplemented with 0.5 mM valproic acid to further increase the efficiency of reprogramming. Medium was changed daily and valproic acid was omitted after seven days. Initial iPSC colonies were picked and further cultivated on 45,000 feeder cells/cm^2^ until a stable cell line was expanded.

### 2.3 Karyotyping

Karyotyping was performed by Giemsa Trypsin banding. Colonies were incubated with colcemid solution (10 µg/ml in HBSS) overnight for metaphase arrest. IPSCs were harvested by trypsin treatment (0.25%), wherein the reaction was stopped with Amniomax solution (Invitrogen, Germany), centrifuged at 300*g* for 10 min and the pellet was resuspended in 4 ml KCl solution (5.62%). Cells were incubated for 5 min at 37 °C and centrifuged at 300*g* for 10 min. After resuspension cells were fixed in 5 ml glacial acetic acid and methanol (1:3) and subsequently centrifuged for 7 min at 350*g*. This step was repeated once. Next, supernatant was removed and cells were resuspended. Cell suspension was dropped onto cold slides and dried at 100 °C for 1 h. Giemsa solution (5%) was added and incubated for 5 min. Slides were washed in distilled water two times, dried at room temperature and sealed with cover slips.

### 2.4 Alkaline phosphatase staining

Alkaline phosphatase (AP) staining used to prove pluripotency of generated iPSCs. Colonies of iPSCs, cultivated on feeder cells, were fixed with ice-cold methanol (100%) for 10 min and incubated at room temperature for 15 min with the staining solution, containing 75% distilled water, 10% sodium chloride solution (1 M), 10% Tris solution (1 M, pH 9.8), 5% magnesium chloride solution (1 M), and NBT/BCIP solution (1:50, Roche, Germany). Microphotographs were taken using a Nikon Eclipse TS100 microscope system (Nikon, Germany).

### 2.5 Immunocytochemistry for pluripotency marker

Cells were fixed at room temperature for 15 minutes in 4% paraformaldehyde, washed with PBS and stored in 0.02% NaN_3_ at 4 °C. Immunocytochemistry was performed for Nanog (1:100, rabbit IgG), Tra-1–60 (1:100, mouse IgM), Tra-1–81 (1:100, mouse IgM), SSEA3 (1:100, rat IgM), SSEA4 (1:100, mouse IgG, all Stemgent, USA). Blocking and permeabilization was carried out using 0.3% Triton X-100 and 5% normal goat serum (Dako, Denmark; in PBS) for 30 min at room temperature. Cells were incubated with primary antibodies for 2 h at room temperature in 1% normal goat serum, followed by three washing steps with PBS. Alexa Fluor 568 (1:500, goat anti-rabbit IgG, Invitrogen, Germany) and Alexa Fluor 488 (1:500, goat anti-mouse IgG or goat anti-mouse IgM or goat anti-rat IgM, Invitrogen, Germany) were used as secondary antibodies, incubated 1 h at room temperature in PBS. Cells were washed three times with PBS and mounted with Mowiol-DABCO mounting medium. Pictures were taken with a Biozero 8000 microscope system (Keyence, Germany).

### 2.6 Generation of embryoid bodies

Embroid body (EB) formation was used for proving pluripotency of iPSCs by using marker for the three germ layers. Whole iPSC colonies were mechanically lifted off the feeder cell layer and transferred into the cavity of a low attachment 6-well plate in 5 ml of differentiation medium (knockout DMEM, 20% FBS, 1% MEM non-essential amino acids, 2 mM GlutaMAX, and 0.1 mM beta-mercaptoethanol). EBs were formed after five to seven days and reseeded onto gelatin-coated glass cover slips. After 10 days of random differentiation, spread cells were fixed with 4% PFA for 15 min and immunocytochemically stained for muscle actin (MA, 1:50, mouse IgG, Dako, Denmark), alpha fetoprotein (aFP, 1:500, mouse monoclonal IgG, Sigma-Aldrich, Germany) and nestin (1:100, mouse IgG, R&D, Germany). Alexa Fluor 488 (1:500, goat anti-mouse IgG (Invitrogen, Germany) was used as secondary antibody, incubated 1 h at room temperature in PBS. Cells were washed three times with PBS and mounted with Mowiol-DABCO mounting medium. Pictures were taken with a Biozero 8000 microscope system (Keyence, Germany). Teratoma formation assay in immune deprived mice was not performed any more, following the 3Rs principle, to replace, refine or reduce, the use of animals, mandated by the German Protection of Animal Act. In regards of the teratoma formation assay in stem cell research the applicability of the *in vitro* embryoid body formation assay is discussed by Sheridan and colleagues [4].

### 2.7 Neural and neuronal differentiation

Neural differentiation of iPSCs was induced by density-dependent growing of the iPSCs on Matrigel (Corning, Netherlands). After neural rosettes had been formed spontaneously, cells were washed with DMEM/F12, singled using Accutase® and then magnetically sorted using magnetic beads against the surface marker PSA-NCAM (Miltenyi Biotec, Germany) which is a marker of the neural lineage. The generated neural progenitor cells were used for 25 passages and seeded at an expansion density of 100,000 cells/cm^2^ on poly-L-ornithine (15 µg/ml; Sigma, Seelze, Germany)/laminin (10 µg/ml; Trevigen, USA)-coated dishes in proliferation medium containing 60% DMEM, 40% DMEM/F-12, 1X B27, 0.5% penicillin/streptomycin, 20 ng/ml FGF2 (Amsbio, United Kingdom), 20 ng/ml EGF (Peprotech, Germany). Pluripotency of neural progenitor cells was proven by stainings for Sox2 (1:200, rabbit IgG, Abcam, United Kingdom), nestin (1:100, mouse IgG, R&D, Germany), Pax6 (1:200, rabbit IgG, Abcam, United Kingdom). Alexa Fluor 568 (1:500, or goat anti-rabbit IgG, Invitrogen, Germany) and Alexa Fluor 488 (1:500, goat anti-mouse IgG, Invitrogen, Germany) were used as secondary antibodies, incubated 1 h at room temperature with 1% normal goat serum in PBS. Cells were washed three times with PBS and mounted with Mowiol-DABCO mounting medium. Pictures were taken with a Biozero 8000 microscope system (Keyence, Germany).

For terminal neuronal differentiation cells were plated at a density of 45,000 cells/cm^2^ in differentiation medium, containing 60% DMEM, 40% DMEM/F-12, 1X B27, 0.5% penicillin/streptomycin, which was changed every 4 days over a period of 6 weeks. Cells were stained for the neuronal marker beta III-tubulin (1:100, mouse IgG Tu-20, Santa Cruz biotechnology, Germany or rabbit IgG, Abcam, United Kingdom), GFAP (1:500, rabbit IgG, Dako, Denmark) and synaptophysin (1:100, mouse IgG, Sigma, Germany). Blocking and permeabilization was carried out using 0.3% Triton X-100 and 5% normal goat serum (Dako, Denmark; in PBS) for 30 min at room temperature. Cells were incubated with primary antibodies for 2 hours at room temperature in 1% normal goat serum, followed by three washing steps with PBS. Alexa Fluor 568 (1:500, goat anti-mouse IgG or goat anti-rabbit IgG, Invitrogen, Germany) and Alexa Fluor 488 (1:500, goat anti-mouse IgG, Invitrogen, Germany) were used as secondary antibodies, incubated 1 h at room temperature with 1% normal goat serum in PBS. After washing with PBS, cells were stained with DAPI (5 min, 250 ng/ml), washed three times and mounted with Mowiol-DABCO mounting medium. Pictures were taken with a Biozero 8000 microscope system (Keyence, Hamburg, Germany).

### 2.8 Patch clamp recordings

Patch clamp recordings were performed using an EPC-10 amplifier (Heka, Germany). Patch pipettes were pulled from borosilicate glass tubing (Harvard Apparatus, USA). The internal solution contained (mM): KCl 130, NaCl 10, HEPES 10, EGTA 11, MgCl_2_x6H_2_O 1, CaCl_2_xH_2_O 2, Mg-ATP 2. pH was adjusted to 7.2. When filled, electrodes had a resistance of 6–8 MΩ. Cell cultures were continuously superfused with an extracellular solution consisting of (mM): NaCl 125, KCl 2.5, CaCl_2_xH_2_O 2, MgCl_2_x6H_2_O 1, NaHCO_3_ 26, NaH_2_PO_4_xH_2_O 1.25, glucosexH_2_O 25. Solution was continuously bubbled with carbogen to maintain a pH of 7.4. Recordings were made in the whole cell configuration with holding potentials (V_H_) of −60 or −80 mV. Current voltage responses were evoked by applying 100 ms voltage steps from −60 mV to +50 mV in 10 mV increments. Current clamp mode was used to apply current steps to induce action potentials or to measure spontaneous action potentials. Postsynaptic currents were measured in the voltage clamp mode at a V_H_ of −60 mV. Mini Analysis 6 (Synaptosoft, USA) was used to analyze recordings of post-synaptic currents.
